# Supplementary material for: A versatile in situ cofactor enhancing system for meeting cellular demands for engineered metabolic pathways
Source: J Biol Chem. 2023 Dec 28;300(2):105598. doi: 10.1016/j.jbc.2023.105598 (PMC10850783; doi:10.1016/j.jbc.2023.105598)
Supplement: Supporting Tables S4–S7 and Figures S1–S8 [file mmc4.pdf]

A versatile *in situ* cofactor enhancing system for meeting cellular demands for engineered metabolic pathways.

Juthamas Jaroensuk[1†], Chalermroj Sutthaphirom[1†], Jittima Phonbuppha[1], Wachirawit Chinantuya[2], Chatchai Kesornpun[1], Nattanon Akeratchatapan[1], Narongyot Kittipanukul[1], Kamonwan Phatinuwat[3], Sopapan Atichartpongkul[4], Mayuree Fuangthong[3,4], Thunyarat Pongtharangkul[5], Frank Hollmann[6], and Pimchai Chaiyen[1*]

[1] School of Biomolecular Science and Engineering, Vidyasirimedhi Institute of Science and Technology (VISTEC); Wangchan Valley, Rayong, 21210, Thailand.
[2] Department of Biochemistry and Center for Excellence in Protein and Enzyme Technology, Faculty of Science, Mahidol University; Bangkok, 10400, Thailand.
[3] Program in Applied Biological Sciences, Chulabhorn Graduate Institute, Bangkok, 10210, Thailand.
[4] Laboratory of Biotechnology, Chulabhorn Research Institute; Bangkok, 10210, Thailand.
[5] Department of Biotechnology, Faculty of Science, Mahidol University; Bangkok, 10400, Thailand.
[6] Department of Biotechnology, Delft University of Technology; Delft, 2629 HZ, Netherlands.

[†]Authors contributed equally

Running title: XR and Lactose synergy for enhancing cofactor synthesis in engineered microbes.

[*]Corresponding author
E–mail: pimchai.chaiyen@vistec.ac.th

List of Supplemental Materials

1. **Supplemental tables: 7**
2. **Supplemental figures and legends: 8**

Supplemental tables

Table S1. (Separate file)
The annotated metabolites of fatty alcohol biosynthesis

Table S2. (Separate file)
The annotated metabolites of bioluminescence light

Table S3. (Separate file)
The annotated metabolites of alkane biosynthesis

Table S4. *E. coli* strains used in this study.

Strain	Characteristics	Source
E. coli BL21 (DE3)	*fhuA2 [Ion] ompT gal (λ DE3) [dcm] ΔhsdS*	Novagen
E. coli BW20767 (ATCC 47084)	RP4-2(tet::Mu-1,kan::Tn*7* integrant) *uidA*(ΔMlu1)::*pir*$^+$ *recA1 creB510 leu-63*::IS*10 hsdR17 endA1 zbf-5 thi*	ATCC
E. coli-far	*E. coli* BL21 (DE3) harboring pETDuet-*mbp-far*	This study
E. coli-far-xr	*E. coli* BL21 (DE3) harboring pETDuet-*mbp-far-gst-xr*	This study
E. coli-far-gdh	*E. coli* BL21 (DE3) harboring pETDuet-*mbp-far-gdh*	This study
E. coli-luxCDEAB	*E. coli* BL21 (DE3) harboring pETDuet-*luxCDEAB*	This study
E. coli-luxCDEAB-xr	*E. coli* BL21 (DE3) harboring pETDuet-*luxCDEAB* and pRSFDuet-*gst-xr*	This study
E. coli-p*fap*	*E. coli* BL21 (DE3) harboring pCDFDuet-*fap* and pRSFDuet-1	This study
E. coli-p*fap*-p*xr*	*E. coli* BL21 (DE3) harboring pCDFDuet-*fap* and pRSFDuet-*gst-xr*	This study
E. coli-fap	*E. coli* BL21 (DE3) harboring pET28a-*fap*	This study
E. coli-fap-xr	*E. coli* BL21 (DE3), *glm*S::pT7-*gst-xr, Gm*^{r} harboring pET28a-*fap*	This study

Table S5. Primers used for plasmid construction.

**Primers**	**Sequence (5'-3')**	**Plasmid template**
F-lacI	GCACGACAGGTTTCCCGAC	pMAL-mbp-far
F-M13	GTAAAACGACGGCCAGT	pMAL-mbp-far
F-gst-xr	ACTTTAAGAAGGAGATATACATGTCCCCTATACTAGGTTATTG	pGEX-gst-xr
R-gst-xr	GTTCGACTTAAGCATTATGCTTAACCAAACAGGTACAG	pGEX-gst-xr
F-luxCDE	ACTTTAAGAAGGAGATATACATGGAAAAACACTTACCTTTAATAATAAATG	pET17b-VcluxCDE
R-luxCDE	CAGGCGCGCCGAGCTCGAATTCGTCAGTTGCCTCCTTCATTCT	pET17b-VcluxCDE
F-luxAB	GTATAAGAAGGAGATATACAATGAAATTTGGAAACTTCCTTC	pET11a-VcluxAB
R-luxAB	CGATCGCGTGGCCGGCCGATTTACGAGTGGTATTTAACG	pET11a-VcluxAB
F-pRSF-xr	GTATAAGAAGGAGATATACAATGTCCCCTATACTAGGTTATTG	pGEX-gst-xr
R-pRSF-xr	TATCCAATTG AGATCTGCCA AATTCTTAACCAAACAGGTAC	pGEX-gst-xr
F-pT7-gst-xr	CACTGGCCGTAAGTGGCGAGCCCGATCTTC	pETDuet-mbp-far-gst-xr
R-pT7-gst-xr	GTGCTGGCGTTTAACCAAACAGGTACAGCTTATTCGC	pETDuet-mbp-far-gst-xr
F-ATG-6XHis-gdh	ACTTTAATAAGGAGATATACATGCACCACCACCACCAC	pRSFDuet-6xHis-gdh
R-ATG-6XHis-gdh	GTTCGACTTAAGCATTATGCTTATCCGCGGCCTGCCTG	pRSFDuet-6xHis-gdh

Table S6. Parameters used for data processing.

**De-multiplexing tool**	
Multiplexing Pulsing Sequence Bit	4 bit
Preprocessing filter level	2-Medium
Filter threshold	100
**IM-MS Reprocessing**	
Recalibrate	
Minimum abundance	As default
m/z tolerance	As default
Reference masses (in Negative *m/z*)	112.985587 and 1033.988109
**IM-MS Browser**	
Reference set	Agilent ESI Tune Mix (neg)
**Mass Profiler**	
Feature Finding/Loading	
Measure of Abundance	Feature volume
Feature finding input filters	Chromatographic data
Restrict RT to	2.0-23.0 min
Ion intensity	≥ 100 counts
Isotope model	Common organic (No halogens)
Limit charge states to the range of	1-2
Alignment and Normalization	
RT tolerance	± 0.5 min
DT tolerance	± 1.5%
Mass tolerance	± (15 ppm + 2 mDa)
RT correction	Apply-without standards
RT correction tolerance	± (0.5% + 0.5 min)
Statistics & Filters	
Q-Score	≥ 50
Restrict m/z to	80.0 – 1100.0
Frequency	≥ 75% (3/4) in at least one group (fatty alcohol project)
	≥ 60% (3/5) in at least one group (bioluminescence light project)
	≥ 75% (3/4) in at least one group (alkane project)

Table S6. Parameters used for data processing (continue).

**Mass Profiler Professional**	
Minimum abundance	≥ 100 counts (follow mass profiler setting)
Minimum ion	≥ 1 (follow mass profiler setting)
Mass range	≤ 1100 (follow mass profiler setting)
RT tolerance	± 0.15 min
DT tolerance	± 1%
Mass tolerance	± (15 ppm + 2 mDa)
RT correction	Apply-without standards
Data transformation	Log2
Normalization	Total abundance
Baselining	Baseline to median

Table S7. Primers used for qRT-PCR.

Primer	Sequence (5'-3')
aceE-F	GATTCTGCAGGAAGGGATCAA
aceE-R	GGAAGCCGAACATCGAGTAATA
cysK-F	CGACGGTCAGGTTGATGTATT
cysK-R	GCTCAACGGCGACAGATATAA
edd-F	GCCGCCACTTGGTGTATTA
edd-R	GCTTCTGGTGTTACGTGGATAG
fbaB-F	GTAGACCGCGTGATGATTGA
fbaB-R	GGTCAACCGGCAGAATAGAA
gntk-F	GCTATGCAGCGCACTAATAAAG
gntk-R	GGTAGATGAAAGAGAGATTCGGATTA
gor-F	CGACGATCAGGTGAAAGTGTAT
gor-R	GCCGTGAATACCGACAATCT
gshB-F	CGTGAAGCAGAACTACGAAGAG
gshB-R	TATCAAACGGCGGGTCTTTAC
hisC-F	AATATCCGCACCTGGCTATTT
hisC-R	CAGGTTGATGACTTCTTCGTTTG
pgi-F	CGTTGGCGAGTTTGGTATTG
pgi-R	TCAAAGCCGATGGAGAGAAC
zwf-F	GGTTTACCGTATCGACCACTATC
zwf-R	GGTGCGATTGTCCCAGTTAT

Supplemental figures and legends

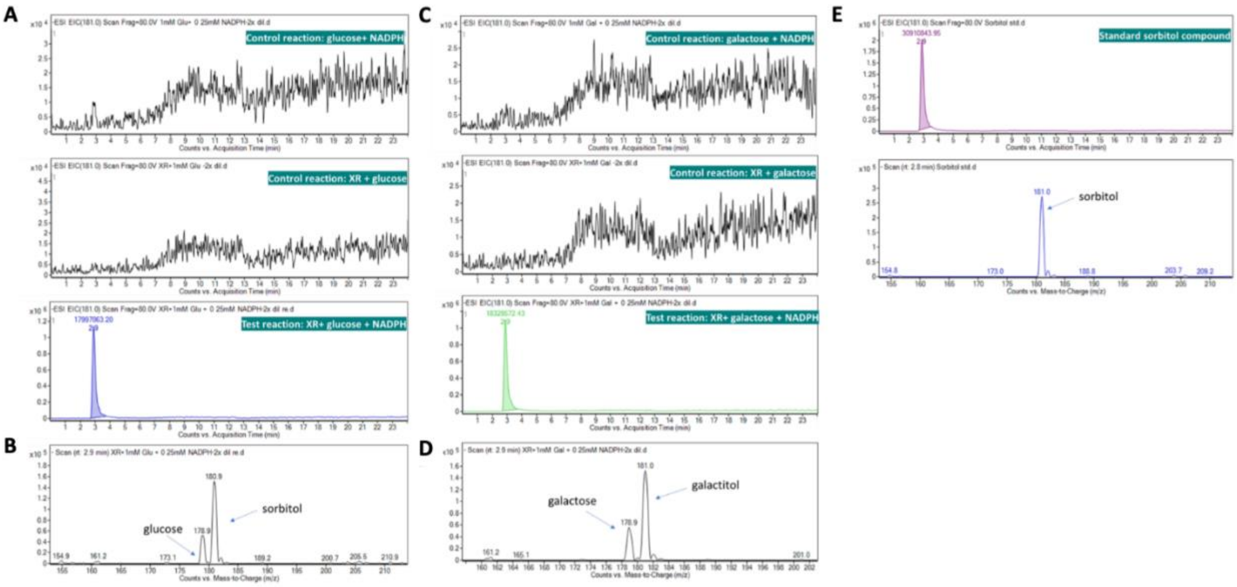

Figure S1. Sugar reduction by XR using D-glucose and D-galactose as substrates. *A-B*, The extracted ion chromatograms (EIC) and exact mass spectra at *m/z* 181.0 ($[M-H]^-$) identified sorbitol as a product from reduction of D-glucose by XR. The reactions were carried out under ambient temperature for 10 min in 100 mM potassium phosphate buffer, pH 7.5 containing 5 µM XR, 0.25 mM NADPH, and 1 mM glucose. The control reaction was performed without addition of XR and NADPH. The sorbitol product was analyzed by an LC-MS triple quadrupole mass spectrometer in negative mode. *C-D*, The extracted ion chromatogram (EIC) and exact mass spectra at *m/z* 181.0 ($[M-H]^-$) identified galactitol as a product from the reduction of D-galactose by XR. The reactions were carried out under ambient temperature for 10 min in 100 mM potassium phosphate buffer, pH 7.5 containing 5 µM XR, 0.25 mM NADPH, and 1 mM galactose. The control reaction was performed without addition of XR or NADPH. The galactitol product was analyzed by LC-MS with a triple quadrupole mass spectrometer in negative mode. *E*, The extracted ion chromatogram (EIC) and exact mass spectra at *m/z* 181.0 ($[M-H]^-$) of a standard sorbitol analyzed by an LC-MS triple quadrupole mass spectrometer in negative mode. The standard compounds and samples were diluted in acetonitrile (ratio 1:1) before being analyzed on an Agilent 1200 series LC 6470 triple quadrupole mass spectrometer. The Agilent Poroshell 120 HILIC-Z 150 ´ 2.1 mm, 2.7 µm (particle size) column was used to achieve optimal separation. The mobile phase was 10 mM ammonium acetate in water pH 9.0 containing 2.5 µM deactivator (mobile phase A) and 10 mM ammonium acetate in 85% ACN pH 9.0 containing 2.5 µM deactivator (mobile phase B) with a flow rate of 0.3 ml/min. The gradient was changed from 4% mobile phase A / 96% mobile phase B to 35% mobile phase A / 65% mobile phase B in 24 min, and the column was maintained at 35˚C. The mass spectrometer was operated in scan mode to detect sugar alcohol products based on their retention times and *m/z* values compared to standard compounds. MS parameters were as follows: gas temperature, 250˚C; gas flow, 12 L/min; nebulizer, 45 psi; sheath gas flow, 12 L/min; capillary voltage, 3000 V; and VCharging, 1000.

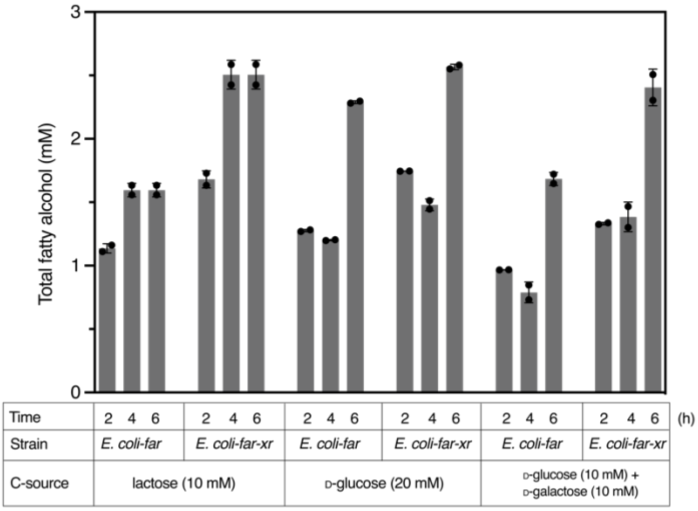

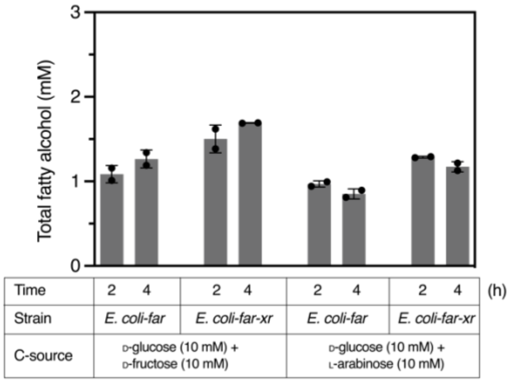

Figure S2. Amount of fatty alcohol (mM) produced by *E. coli-far* and *E. coli-far-xr* at various time points of bioconversion using various types of sugars. *E. coli-far* and *E. coli-far-xr* were induced with 1 mM lactose at 220 rpm, 25˚C for 6 h and then harvested and employed as biocatalysts for fatty alcohol production. The bioconversion reactions were carried out in 0.1 M potassium phosphate buffer at pH 7.5 containing cells with OD_{600} of 30/ml and 10 mM lactose, 20 mM D-glucose, 10 mM each of D-glucose/D-galactose, 10 mM each of D-glucose/D-fructose, and 10 mM each of D-glucose/L-arabinose. The reactions were incubated at 220 rpm, 25˚C for 2, 4, and 6 h with 10 mM each of D-glucose/D-galactose, 20 mM D-glucose, and, and for 2 to 4 h for 10 mM each of D-glucose/D-fructose and 10 mM each of D-glucose/L-arabinose. Fatty alcohol products were extracted with 2 ml of ethyl acetate containing internal standard (200 µM of tetradecane). Data are shown as mean ± s.d., n = 2 replicate cultures. The addition of XR clearly improved the yield of fatty alcohol production by the whole-cell biocatalysts with all tested sugars, particularly with mixed D-glucose/D-galactose and lactose utilization. This might be due to XR conversion of glucose into its corresponding alcohol (sorbitol), alleviating the glucose repression effects and allowing the cell to co-utilize other carbon sources for creating the necessary precursors simultaneously for fatty alcohol synthesis. It should be noted that when D-glucose was used as a sole substrate, the enhancement effect in production of fatty alcohol by *E. coli-far-xr* compared to *E. coli-far* was less than that seen for the conditions using lactose, D-glucose with D-galactose, D-glucose with D-fructose, or D-glucose with L-arabinose. The data imply that XR can enhance fatty alcohol production greater in the presence of mixed carbohydrate substrates.

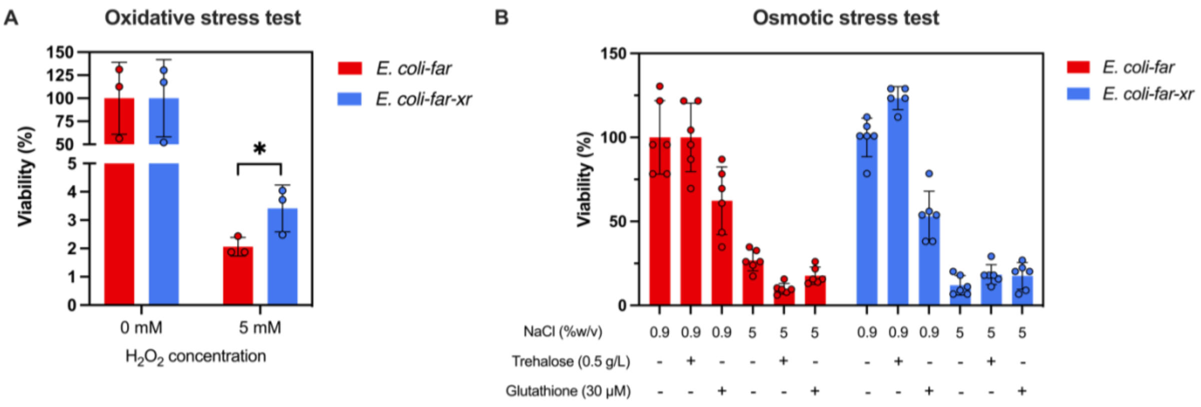

Figure S3. Oxidative and osmotic stress test on *E. coli-far* and *E. coli-far-xr*. *E coli* strain FAR and FAR-XR protein overexpression was induced with 1 mM lactose for 6 h and used for an oxidative and osmotic stress assays by adding H_2O_2 and NaCl, respectively. *A*, A 0.5-ml reaction contained the biocatalyst with final OD_{600} of 0.4/ml and 5 mM H_2O_2 in 0.1 M Kpi pH 7.5. After incubation for 90 min at room temperature, the viable cells were measured by colony forming unit (CFU) counting on an LB agar plate. The results suggested that the cells containing XR had higher CFUs than the cells without XR. Data are shown as mean ± s.d., n = 3 replicate cultures. Asterisk indicates significant differences by *t* test ($p < 0.05$). *B*, A 0.5-ml reaction contained biocatalyst with a final OD_{600} of 0.4/ml, NaCl, trehalose (0.5 g/L), or glutathione (30 μM) in 0.1 M Kpi pH 7.5. After incubation for 3 h at room temperature, the number of viable cells were measured by colony forming unit (CFU) counting on an LB agar plate. Most of the results for the two cell types were similar except that *E. coli-far* showed a higher percentage of cell viability than *E. coli-far-xr* in the presence of 5% (w/v) NaCl (without addition of trehalose or glutathione) ($p < 0.05$, *t* test, Bonferroni-Dunn method). The results suggest that the up-regulation of trehalose and glutathione found in *E. coli-far-xr* does not enhance resistance to osmotic stress. Data are shown as mean ± s.d., n = 5 replicate cultures.

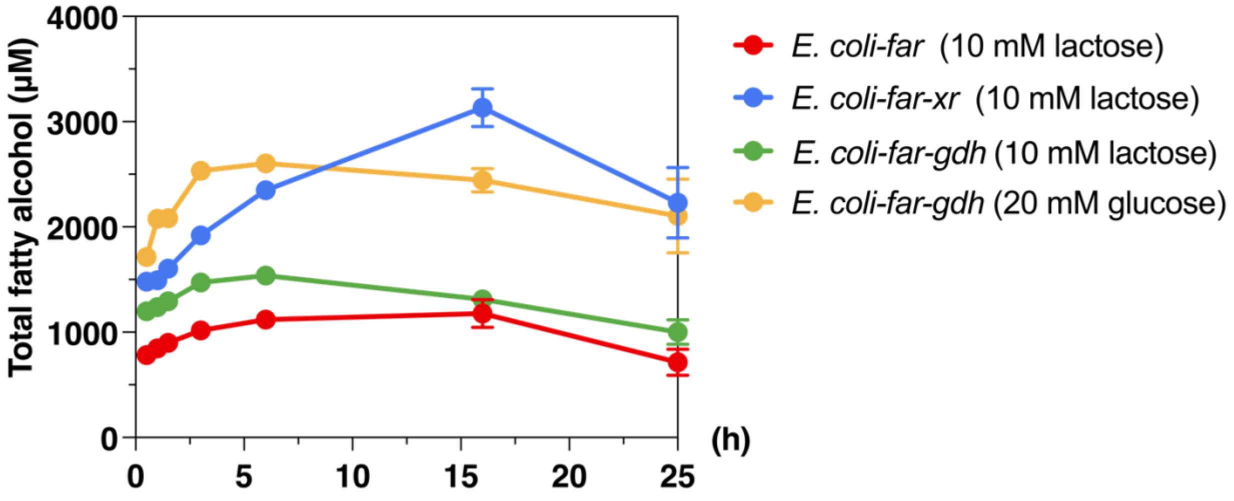

Figure S4. Comparison of the fatty alcohol production in the bioconversion by different cofactor regenerating systems in the engineered cell. The bioconversion was described in Experimental procedures. Fatty alcohol was detected during the bioconversion in *E. coli-far* (red), *E. coli-far-xr* (blue), *E. coli-far-gdh* when 10 mM lactose was used as a substrate (green) and when 20 mM D-glucose was used as a substrate (orange) in *E. coli-far-gdh*. Analytical methods were described in Experimental procedures. Data are shown as mean ± s.d., n = 3 replicate cultures.

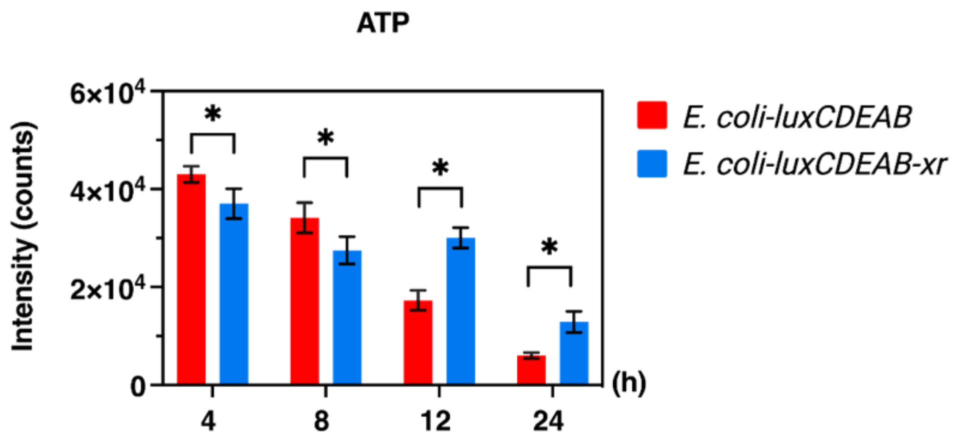

Figure S5. Time-course analysis of ATP in *E. coli-luxCDEAB* and *E. coli-luxCDEAB-xr* after adding 10 mM lactose for 4, 8, 12, and 24 h. The samples were diluted in acetonitrile (ratio 1:1) before being analyzed by the LC 6470 triple quadrupole mass spectrometer (Agilent 1200 series). An Agilent Poroshell 120 HILIC-Z 150 ´ 2.1 mm, 2.7 µm (particle size) column was used to achieve optimal separation. The flow was 0.3 ml/min with a mobile phase of 10 mM ammonium acetate in water pH 9.0 containing 2.5 µM deactivator (mobile phase A) and 10 mM ammonium acetate in 85% ACN pH 9.0 containing 2.5 µM deactivator (mobile phase B). The gradient was changed from 4% of mobile phase A / 96% of mobile phase B to 35% of mobile phase A / 65% of mobile phase B over 24 min., and the column was maintained at 35˚C. The mass spectrometer was operated in a SIM mode to detect ATP based on the parameters of retention time and *m/z* by comparison to values of the standard compound. MS parameters were as follows: gas temperature, 250°C; gas flow, 12 L/min; nebulizer, 45 psi; sheath gas flow, 12 L/min; capillary voltage, 3000 V; and VCharging, 1000. Data are shown as mean ± s.d., n = 5 replicate cultures; error bars show s.d; asterisk denotes significant differences by multiple *t* test ($p < 0.05$).

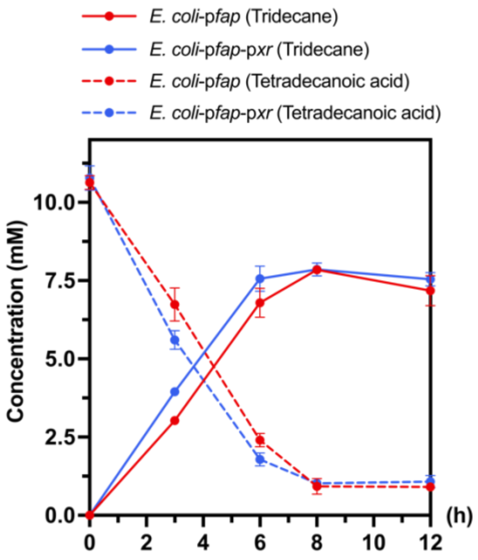

Figure S6. Tridecane production (mM) from tetradecanoic acid by *E. coli* harboring FAP with/without XR overexpressed in the plasmid system. Plasmids of pCDFDuet-*fap* and pRSFDuet-*gst-xr* (pRSFDuet in case of biocatalyst without XR) were co-transformed into *E. coli* BL21 (DE3) to generate *E. coli*-p*fap* and *E. coli*-p*fap*-p*xr*. A single colony of the resulting transformants was inoculated in LB broth in the presence of streptomycin 25 µg/ml and kanamycin 34 µg/ml at 220 rpm, 37˚C for 17 h. The starter cultures were then sub-cultured with 1% inoculant into TB media containing a half-dose of the antibiotics mentioned above and grown at 37˚C until the OD_{600} reached 0.6-0.7. The cultures were then induced with 10 mM lactose and incubated further with shaking at 220 rpm, 25˚C for 12 h, then harvested and employed as biocatalysts for alkane production. The bioconversion reactions were carried out in potassium phosphate buffer (0.1 M, pH 7.0) containing a cell biocatalyst at an OD_{600} of 60/ml and 10 mM tetradecanoic acid. The reaction was set in a capped 20 ml clear glass vial and incubated under blue light (PPFD-B 20 µmolphotons/m^2/s) with shaking at 100 rpm, 25˚C. Fatty acid and alkane were extracted using 4 ml of ethyl acetate containing the internal standard (200 µM of tetradecane) and analyzed by GC-FID equipped with HP-5 column. Data are shown as mean ± s.d., n = 3 replicate cultures. Three hours after the reaction was initiated, the productivity of *E. coli*-p*fap*-p*xr* (blue) was 1.3 mmol/L/h, which is higher than 1 mmol/L/h of *E. coli*-p*fap* (red) by 1.3-fold. To improve the use of the XR/lactose system as an enhancer system for producing the active FAP (FAP:FAD) without adding extra antibiotic and creating a more stable biocatalyst, we further created a strain of *E. coli* BL21 (DE3) with an XR integrated genome (see construction details in Experimental procedures). The use of the XR genome integrated cell version for production of tridecane was discussed in the Main text.

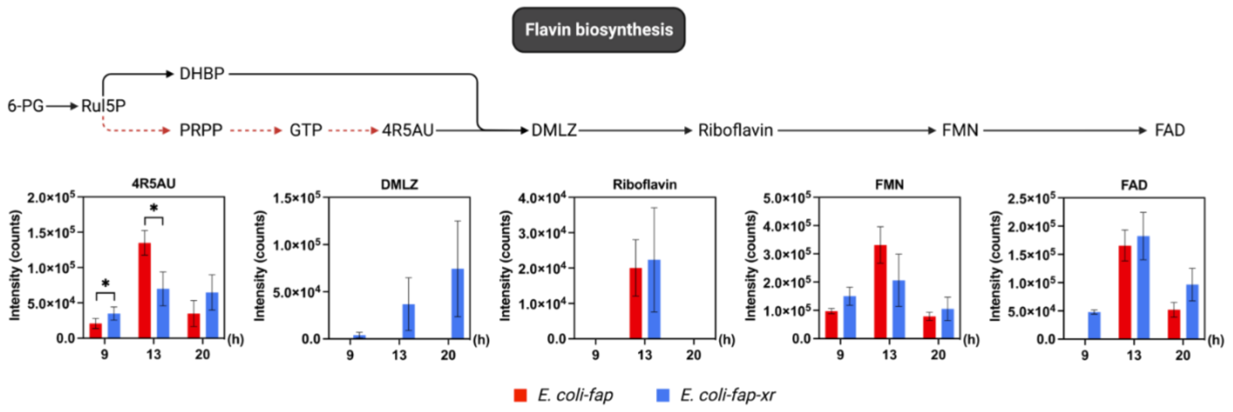

Figure S7. Level of intermediates in flavin biosynthesis during protein overexpression of *E. coli-fap* and *E. coli-fap-xr*. After 75 mM of lactose was added into the TB culture of *E. coli-fap* and *E. coli-fap-xr*, the cells (at equivalent cell amounts based on OD_{600} in 1 ml) were taken from the culture at 9, 13, and 20 hours. The metabolites were extracted from the samples and analyzed by LC-IM-QTOF as described in the Experimental procedures. The levels of metabolites were obtained from the peak area by Find by the Formular (FBF) function with mass tolerance ±30 ppm in the MassHunter Qualitative software (Agilent), with the exception of DMLZ, for which levels were obtained from the abundance value as identified by Mass Profiler. Data are shown as mean ± s.d., n = 6 technical replicates (2 biological replicates), and the asterisk denotes a significant difference by multiple *t* test ($p < 0.05$, Bonferroni-Dunn method).

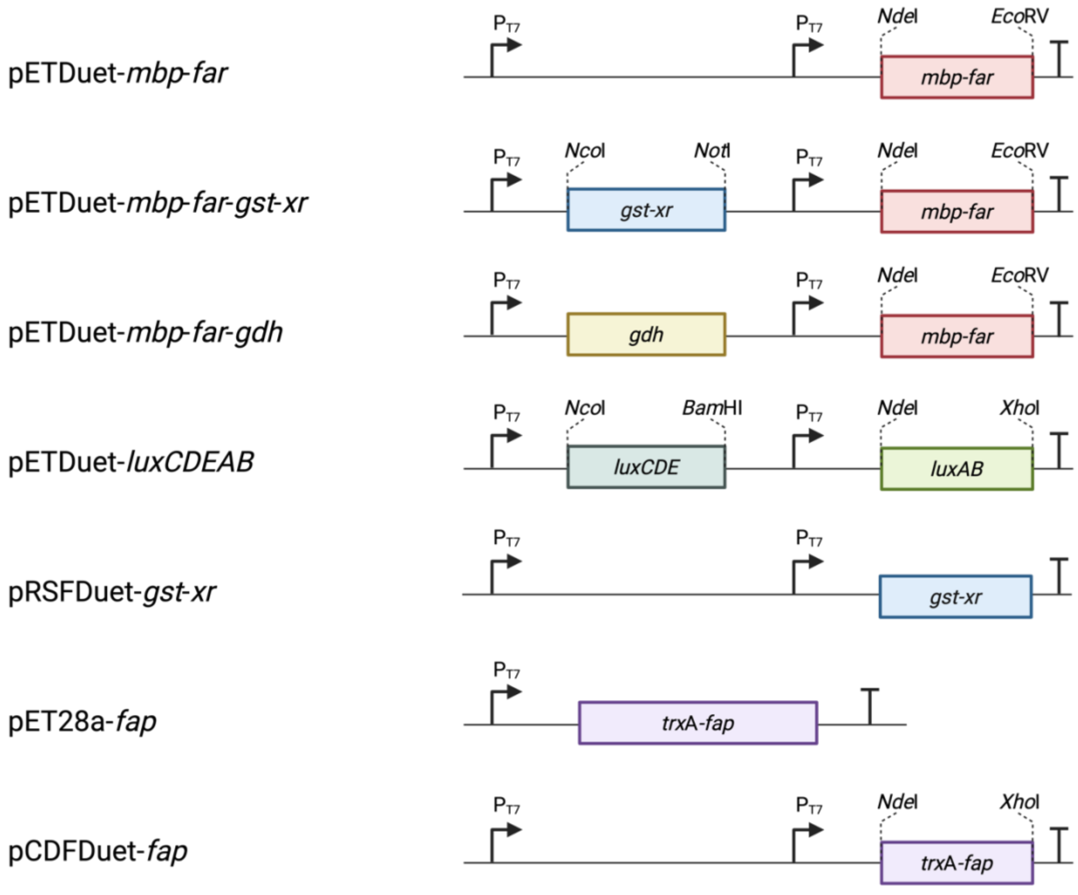

Figure S8. Expression plasmid design used in this study. Details of construction are described in Experimental procedures; P_{T7} = T7 promoter.
